# Deciphering neural heterogeneity through cell lineage tracing

**DOI:** 10.1007/s00018-020-03689-3

**Published:** 2020-11-05

**Authors:** María Figueres-Oñate, Rebeca Sánchez-González, Laura López-Mascaraque

**Affiliations:** 1 Department of Molecular, Cellular and Development Neurobiology, Instituto Cajal-CSIC, 28002 Madrid, Spain; 2 Present Address: Max Planck Research Unit for Neurogenetics, 60438 Frankfurt am Main, Germany

**Keywords:** Clonal analysis, Neural stem cell, Progenitor potential, Cell progeny, Ontogeny, Cell heterogeneity

## Abstract

Understanding how an adult brain reaches an appropriate size and cell composition from a pool of progenitors that proliferates and differentiates is a key question in Developmental Neurobiology. Not only the control of final size but also, the proper arrangement of cells of different embryonic origins is fundamental in this process. Each neural progenitor has to produce a precise number of sibling cells that establish clones, and all these clones will come together to form the functional adult nervous system. Lineage cell tracing is a complex and challenging process that aims to reconstruct the offspring that arise from a single progenitor cell. This tracing can be achieved through strategies based on genetically modified organisms, using either genetic tracers, transfected viral vectors or DNA constructs, and even single-cell sequencing. Combining different reporter proteins and the use of transgenic mice revolutionized clonal analysis more than a decade ago and now, the availability of novel genome editing tools and single-cell sequencing techniques has vastly improved the capacity of lineage tracing to decipher progenitor potential. This review brings together the strategies used to study cell lineages in the brain and the role they have played in our understanding of the functional clonal relationships among neural cells. In addition, future perspectives regarding the study of cell heterogeneity and the ontogeny of different cell lineages will also be addressed.

## Introduction

One fundamental issue in Neuroscience is how the different lineages in the brain are established and what contributions sibling cells make to the nervous system and how they influence its behavior. The current belief is that there is large cell heterogeneity in the adult brain, raising the question as to how these different cell types are generated during development. However, a fundamental question is whether this heterogeneity is ontogenically determined and if so, what are the physiological implications of this? Thus, lineage tracing has developed from the need to pursue all the progeny of specific neural progenitor cells (NPCs) to determine how complete neural networks are built and the contribution of specific progenitors to these networks.

### Neural stem cells potential and heterogeneity

**Fig. 1:**
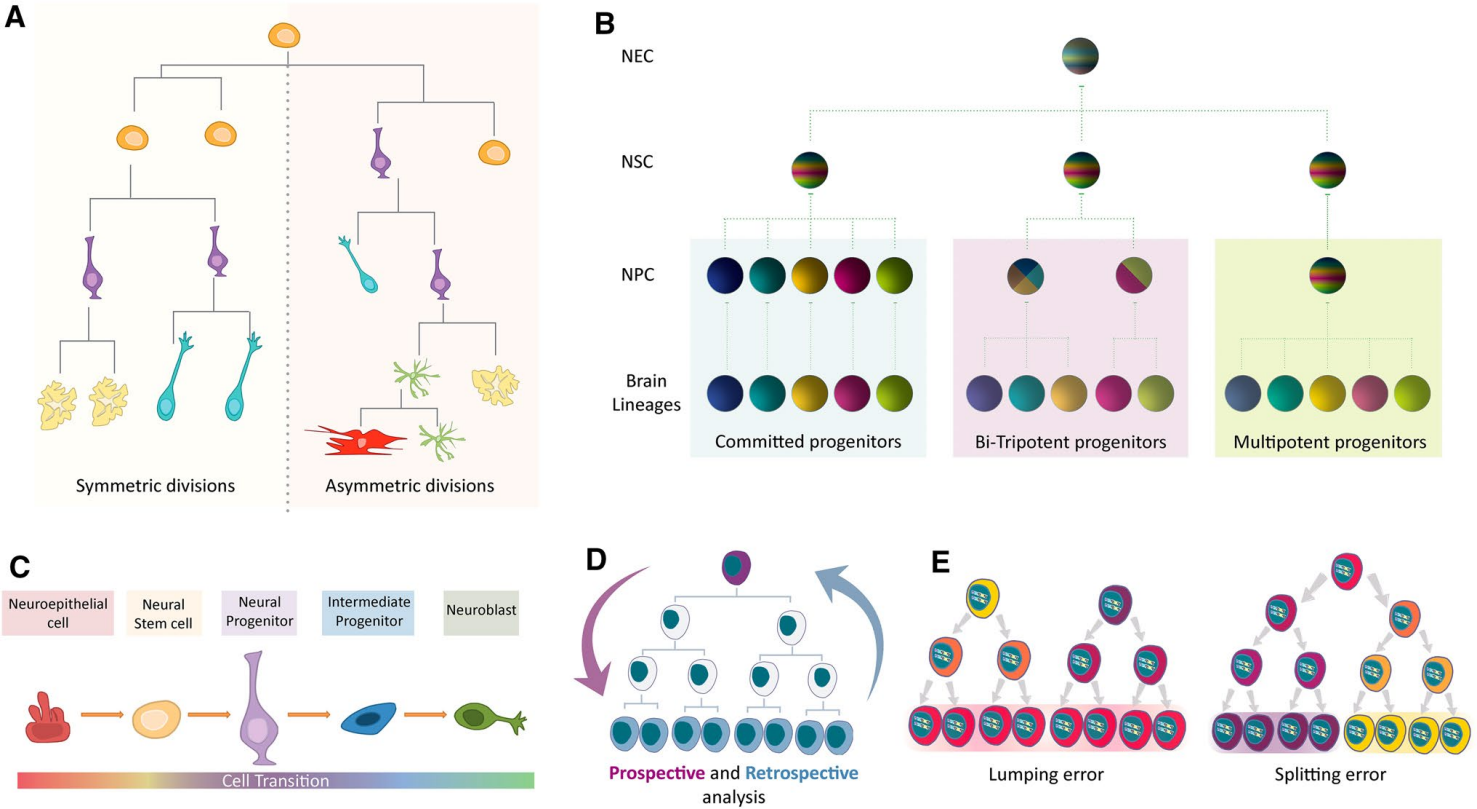
**a** Lineage specification of stem cells throughout development occurs through symmetric and asymmetric divisions. **b** Scheme of the different progenitor potentials of the neuroepithelial cells (NECs) that may coexist in the developing brain. Committed progenitor cells can only give rise to one neural cell type, whereas multipotent progenitors can contribute to all lineages. **c** Graphical representation of the differentiation of NECs to neuroblasts. The gradual maturation of these cells is reflected in the morphological and molecular changes accompanying their lineage transitions. **d** Scheme of the prospective (pink) and retrospective (blue) reconstruction lineage approaches. Prospective lineage tracing targets specific progenitors to trace their progeny, whereas retrospective analyses reconstruct the lineage tree from the descendants to the progenitor. **e** Lumping errors are the result of considering non-clonally related cells as part of the same clone, whereas splitting errors take place when sibling cells are considered as independent clones due to methodological shortcomings. *NEC* neuroepithelial cell, *NSC* neural stem cell, *NPC* neural progenitor cell

Neural stem cells (NSCs) are cells that self-renew and that can produce all the lineages present in the adult brain [1]. Thus, the cell diversity in the brain emerges as the progeny of NSCs progress into lineage-restricted NPCs, more committed cell populations with a more limited differentiation and proliferation potential [2]. The transition to a specific lineage and the consequent loss of potential takes place through symmetric or asymmetric cell divisions. Symmetric divisions amplify the pool of progenitors, generating two identical siblings, whereas asymmetric divisions generate two different daughter cells one of which at least will be more committed to a certain lineage (Fig. 1a).

At early embryonic stages, the principal neural lineages are specified in the neural plate, a defined region of the ectoderm. In parallel, cells of other origins colonize the prospective brain, such as microglia (mesoderm) and blood vessel cells (endoderm). Initially, NSCs known as neuroepithelial cells (NECs) undergo symmetric cell divisions to amplify their pool and prior to generating bipolar radial glial cells (RGCs) [3]. These RGCs produce all the major cell types in the brain and they are often considered the NSCs of the developing brain [4]. RGCs can either proliferate symmetrically to maintain their pool or asymmetrically to generate intermediate progenitor cells (IPCs). RGCs or IPCs will then divide to generate neuroblasts, post-mitotic cells that are committed to generate mature neurons, or glial cells such as oligodendrocytes, astrocytes or NG2 cells [5]. RGCs form a complex cell population that displays region-specific gene expression in the developing nervous system [6]. NSCs are mainly located in the sub-ventricular zone (SVZ), a neurogenic niche that covers different microdomains of the ventricular embryonic walls comprising the pallium, sub-pallium and septum [7]. The neurogenic niche is considered as a heterogeneous pool of cells, with multiple progenitor types displaying either stem cell attributes or more restricted fates [8]. For example, multipotent NSCs that are capable of giving rise to all brain lineages may exist alongside tri- or bi-potent progenitors that have a more restricted potential [9–11]. NPCs that will contribute only one neural cell type could also be present in these neurogenic niches [12, 13] (Fig. 1b). In the brain, cell specification commences with neurogenesis, whereby neuroblasts give rise to the new neurons. Thereafter, astrogenesis occurs and ultimately, oligodendrogenesis commences that continues throughout perinatal stages [14]. This progression is regulated by intrinsic changes in gene expression but also, it is determined by interactions with environmental and developmental cues. Furthermore, the switch from neurogenesis to gliogenesis is driven by competition between downstream transcription factors and growth factor signaling [15, 16]. Different patterning genes are responsible for boundary formation, events that can determine the identity and fate of the NPCs in diverse domains along the lining of the ventricular surface [3].

New approaches in single-cell transcriptome technology have provided novel data regarding cell heterogeneity, addressing the varied gene expression in different brain populations. Specifically, a compilation of molecular markers has been described in the neurogenic niche, expressed in either active or quiescent NPCs under physiological conditions and after brain insult [17–19]. In these studies, cells from the SVZ have been identified based on the expression of particular antigenic markers. Thus, the identity and dynamics of NPCs have been assessed by considering the whole population of ventricular progenitors that express a specific combination of different markers. However, cells expressing selected markers but that are isolated from different areas display distinct self-renewal and differentiation capacities, and they have a distinctive gene expression profile [20]. Thus, no single molecular marker can unambiguously define separate populations. Lineage transition between NPCs and their progeny is achieved by gradual cell maturation, and thus, the expression of the same molecular markers in diverse cell types may overlap at distinct moments in their maturation (Fig. 1c). In the adult neurogenic niche of the SVZ, NSCs have classically been defined as GFAP (Glial Fibrillary Acidic Protein) expressing cells with a particular morphology [21], although this protein is also expressed by the surrounding astrocytes [22]. Furthermore, CD133 (or prominin1) is a transmembrane glycoprotein expressed by the cilia of the adult NSCs in the SVZ [23], and it is also a molecular marker expressed in ependymal cells populating the same surface [24]. Nestin has been considered to be a typical marker to identify NSCs, although it is still expressed in the IPC population [25]. Another molecular marker used to identify embryonic and adult NSC populations is CD24, which is also expressed in some neuroblast populations, albeit at different levels [26]. Thus, cell diversity studies should be based on the potential of single progenitors rather than that of NPCs pools, requiring finer analyses at the individual cell level.

Together, defining NPC populations using specific markers has some limitations, as it is often impossible to precisely define these complex and heterogeneous cell populations solely in this manner. Accordingly, clonal analysis and lineage tracing has become crucial to further understand the biology of NSCs and their lineage progression [27, 28].

### The onset of lineage tracing

Due to the large heterogeneity within the neural progenitor pool, clonal analysis is particularly important when studying stem cell biology. This concept arose at the end of the nineteenth century with the studies of Whitman and collaborators on leech embryos [29], and it is still the subject of intense study today. Cell lineage tracing facilitates the definition of ontogeny, fate and cell behavior in specific tissues or organisms. In the brain, lineage tracing allows the cell progeny of a single neural progenitor to be identified and tracked, and the clonal relationships among co-habiting cells to be defined in the adult. Labeling all the progeny of a specific progenitor or cell population may reveal specific patterns of progenitor proliferation, migration and differentiation. Different prospective and retrospective methodologies have been developed to study cell lineages in the brain (Fig. 1d). Prospective lineage tracing strategies require an initial population of interest to be targeted that will be followed over time, whereas retrospective analysis aims to reconstruct lineage trees from the descendants based on a non-bias selection of the progenitors. The principal drawback when designing clonal methods are the potential lumping and splitting errors (Fig. 1e). Lumping errors arise from considering cells generated from different progenitors as part of the same clone. By contrast, cells that are part of the same lineage could be treated as non-sibling cells due to methodological issues, leading to splitting errors.

Some of the first complete lineage tracing studies were achieved by direct microscope observation of lineage progression using either organisms with a small number of cells, or by isolating NPCs and tracking their divisions in vitro [30]. A ground breaking achievement in the field was the tracing of the entire lineage tree of the nematode *C. elegans* [31] by time-lapse microscopy. However, direct observation in vivo is not usually viable due to the opacity of the tissue or the aim of studying a larger organism. Nevertheless, direct observation of embryonic development in vivo through an intravital window can be combined with different cell labeling approaches, and this has permitted the monitoring, manipulation and live imaging of mouse embryos [32]. The incorporation of non-toxic chromogens into living cells, referred to as vital dyes, was one of the first methods used to visualize cells over time. The principal advantage of fluorescent vital dyes is that they are easily administered in vivo and they do not require post-processing to be visualized. Among the initial fluorescent dyes widely used were the fluorescent retrograde markers like Fast Blue (a cytoplasmic marker) and Diamidino Yellow (a nuclear marker). These dyes not only produced retrograde labeling of groups of cells but they could also be injected simultaneously to detect double labeled cells [33]. In addition, lipophilic carbocyanine fluorescent dyes like DiI and DiO have been used for anterograde and retrograde neuronal tracing in vivo, and in fixed tissue [34, 35], offering a detailed view of the cell’s morphology. The labeling of proliferative cells by incorporating a nucleoside analog like 5-Bromo-2′deoxyuridine (BrdU) can be also used to study cell lineage and fate potential [36, 37] However, a drawback in these approaches is the dilution of the tracer, triggering the consecutive loss of labeling that is most evident in actively proliferating cells [38].

The discovery of the green fluorescent protein (GFP) [39] and of β-galactosidase encoded by the *E. coli* LacZ gene [40] revolutionized lineage tracing, taking over from the use of vital dyes. Reporter genes encoding these proteins were introduced to target cells by lipofection [41] or electroporation [42], and viral particles carrying these reporters could infect cells, integrating the desired recombinant DNA into the host genome to allow cell tracing [43]. Retroviruses have been used widely to trace cell lineages [44–46]. The reporter genes are introduced via retroviral vectors that integrate into the genome of dividing target cells and they are consequently transferred to all their progeny. The first viral tracing methods focused on targeting a limited number of sparse progenitor cells to ensure that the labeled cells populating the same area would pertain to the same clone. Serial dilution of the viral particles can be used to target fewer isolated progenitors. However, an increment of the number of traceable lineages and a stronger lineage analysis was obtained using retroviral libraries that include DNA barcoding [47]. These barcodes can be read after cell sorting or laser dissection, permitting a reconstruction of the lineages. Nevertheless, the use of retroviral labeling can be compromised by epigenetic silencing and the inability to transfect quiescent or non-mitotic cells [48].

Tracking cells from their progenitors to their final destination and fate has been made possible through the development of some important genetic tools that have made permanent cell labeling feasible. In particular, the Cre-LoxP [49] and Flp-FRT [50] recombination systems represented an important advance to study cell progeny. The incorporation of a tamoxifen-inducible version of this technology in transgenic mouse lines expanded the possibilities of undertaking cellular studies in genetically modified organisms (GMOs) [51]. Transgenic mouse lines that are susceptible to inducible-Cre recombination under the control of specific promoters can drive the expression of a fluorescent reporter (FR) to fate map neural progenitors in vivo [52]. This has been achieved by administering low doses of tamoxifen and depending on the lineage of interest, transgenic mice encoding different FRs under the control of specific promoters have been generated, exhibiting particular advantages and disadvantages [53].

In addition, early lineage tracing studies used chimeric mice generated from tetraparental embryos enabled the origin of many structures in the body to be determined [54]. Moreover, transplanting labeled cells from GMOs into wild type animals has also been used to trace cell lineages [55, 56], although one shortcoming of this approach is that the grafted cells may not behave as they do under physiological conditions [57].

### Multicolor lineage tracing

**Fig. 2:**
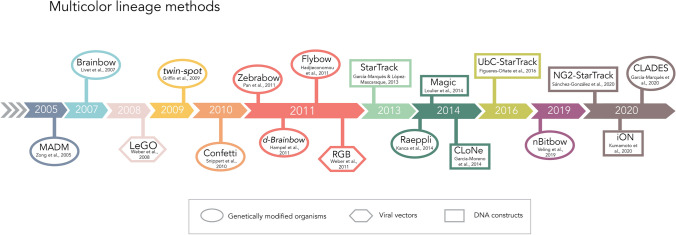
Timeline of multicolor lineage methods. Diagram of the most relevant approaches using combinations of fluorescent reporter proteins for fate mapping. The different strategies are represented by a circle for genetically modified organisms like mice, zebrafish or Drosophila. A hexagon shows the strategies based on recombinant viral particles, and the square represents those approaches involving the use of DNA constructs encoding the different fluorescent reporters

The employment of GMOs and the incremental growth of reporters led to the idea of combining different fluorescent proteins to track sibling cells (Fig. 2). Initially, a few fluorescent variants of GFP were combined to stain neurons individually [58] but undoubtedly, the true revolution in multicolor lineage methods commenced with the appearance of the *Brainbow* technology [59]. Brainbow transgenic mice undergo stochastic recombination of up to four FRs that are driven by the Cre-LoxP system, giving rise to multicolor mosaics in which single cells can be easily identified. Modified versions of this methodology are still being developed [60, 61] and they produce vivid images, that allow an extremely detailed visualization of the morphologies of individual labeled cells. Hence, this is a very effective method for cell mapping but not for lineage tracing. However, this technology has had a tremendous impact on the field of lineage tracing, leading to a new wave of methods in which the stochastic combination of FRs is used to generate unique barcodes in NSCs that can be inherited by all their progeny. This method was originally designed for mice, although the combinatorial use of fluorescent proteins has been remodeled to fate mapping in *Drosophila melanogaster* (Flybow, d-Brainbow, Raeppli) [62–64] and Zebrafish (Zebrabow) [65, 66]. The nuclear expression of more than one fluorophore per cell has generated new modifications of the Brainbow-like technique in *Drosophila* (nBitbow) [67]. Other clonal methods isolate recombination in stochastic cells involving different transgenic lines, such as the mosaic analysis with double markers (MADM) [68, 69], and this technology has also been modified to *D. melanogaster* (twin-spot) [70]. The principal drawback of these techniques, besides the requirement for GMOs, is the small range of combinations to accurately define daughter cells, and the susceptibility to clonal splitting or lumping errors. Moreover, visualizing the fluorescent signal in some of these transgenic mice requires immunostaining [28], an important limitation due to the lack of antibodies to specifically recognize these FRs. Thus, to extend multicolor lineage tracing to different organisms or even to in vitro assays, recombinant viral particles or DNA plasmids have been designed. Recombinant lentiviruses encoding different FRs have been generated to contribute to the multicolor mosaic for lineage tracing (LeGo) [71, 72]. New approaches involve recombinant DNA constructs that encode different FRs, and that can be transfected into the cells of interest, have allowed all the progeny of single cells to be traced (StarTrack, CLONE, MAGIC, iON) [73–76]. To resolve the timing of the birth of each cell within the same clone, lineage progression could be determined post hoc by the expression of a predetermined sequence of fluorophores in sibling cells in *Drosophila* (CLADES) [77]. These methods are based on transposable elements that are integrated into the genome, allowing the stable inheritance of the same barcode by all cell progeny, and avoiding plasmid loss as a consequence of cell division. The combinatorial use of integrable FRs does not require post-processing to visualize the signals due to the bright and stable expression of these proteins, thereby representing an accessible and convenient technique to use in vivo. Moreover, injection of the reporter proteins in vitro or in vivo facilitates the targeting of progenitor cells at a single-cell level, avoiding the need to produce GMOs. New advances in microscopy have produced progress in multicolor lineage tracing [78], expanding the possibility of performing lineage tracing in any organism and lineage, and enabling studies of cell heterogeneity.

Our laboratory has developed a stochastic clonal analysis method called *StarTrack*, which allows the progeny of single cells to be traced and analyzed thereafter. The StarTrack methodology was an attempt to develop a genetic in vivo lineage-tracing method that could track all the neural progeny of individual GFAP cells [73]. It is based on the transfection of cells (by electroporation) with a combination of recombinant DNAs encoding six different FRs that are expressed in different cell compartments (cytoplasm or cell nucleus). This produces inheritable marks that permit the long-term in vivo tracking of the different neural cells generated during embryonic development to their final fate in the adult brain. Due to the use of an ubiquitous promoter, the UbC-StarTrack methodology enables the progeny of embryonic and postnatal neural progenitors to be tracked in mice, irrespective of their fate [79]. Recently, to specifically track the descendants of NG2 progenitors, the promoter of either the transposase or transposon constructs was adapted accordingly [80, 81]. Novel methods based on vector integration show high efficiency in terms of stable integration and the transmission of unique fingerprints to their descendants, which can be followed through several cell divisions and along different lineages through postnatal or adult stages. Furthermore, these strategies can be extrapolated to perform clonal and functional analyses in different animal models or in vitro assays. The increase in the number of FRs expressed in different compartments augments the number of possible combinations available in these integrable multicolor methods. For example, the use of six different fluorophores in two different cell compartments (e.g., nuclear and cytoplasmic) leads to a total of 4095 possible color codes. In addition, the number of copies of each reporter could be resolved by analyzing the intensity of fluorescence in each cell [79], helping to minimize possible splitting and lumping errors.

In summary, multicolor image-based lineage tracing methods are among the most reliable methods to define lineage trees. By combining them with state-of-the-art approaches like cell type-specific optogenetic manipulations, single-cell transcriptomic analysis, cell ablation, live-cell imaging, patch-clamp recordings, two-photon microscopy, in vivo and brain slice preparations, they may help us to better understand how lineages are derived in the brain. In addition, they provide a more functional readout of the specific characteristics of clonally related cells derived from the same NPC, adding information regarding their spatial relationships.

### Single-cell sequencing and CRISPR to address cell lineage tracing

Next-generation sequencing (NGS) provides us with a powerful tool to sequence the whole genome and it opens a window to complete the study of whole organisms, elucidating their genomic, transcriptomic, epigenomic and proteomic profiles. NGS contributes to lineage tracing through both whole genome sequencing and whole-exome sequencing. It has revealed somatic mutations that accumulate in cells after replication that can be traced to define lineage progression [82]. These analyses focused on individual cells, revealing the intricate cell heterogeneity at the single-cell level. Single-cell RNA sequencing (scRNA-seq) is one of the most powerful tools to identify patterns of cell expression and intrinsic molecular profiles. However, single-cell transcriptomic analysis lacks information regarding ontogenic and spatial tracing. Genomic barcoding using viral libraries, followed by the isolation and sequencing of single cells, has facilitated the fate mapping of neural cells, helping to understand their ontogeny and their lineage potential, and complementing classical barcoding methods [83, 84]. Similarly, the combination of classic barcoding techniques and scRNA-seq could help understand the complex biological systems underlying physiological events and pathological conditions.

**Fig. 3:**
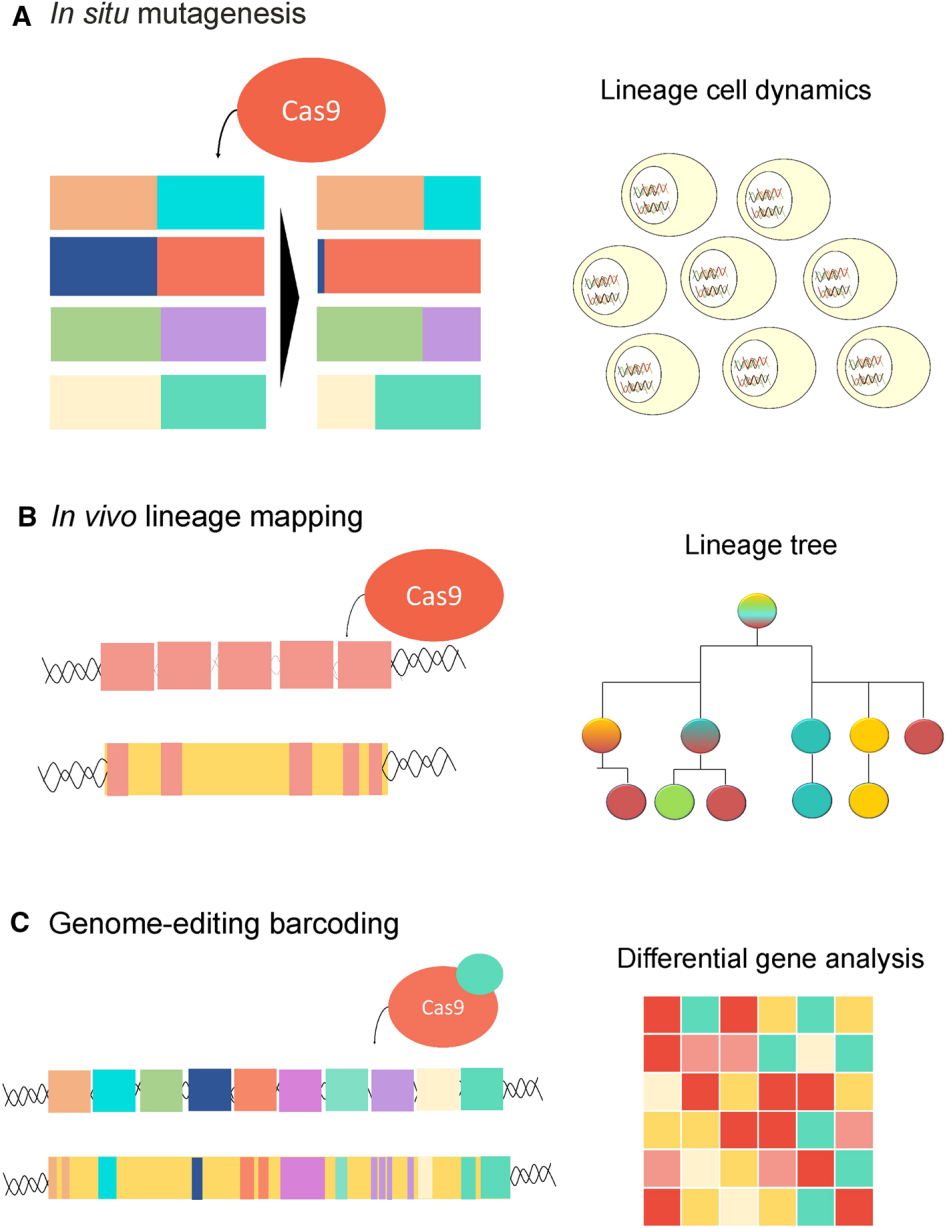
Timeline of the use of CRISPR/Cas9 for lineage tracing. **a** In situ mutagenesis involved the accumulation of multiple integration target sites (called barcoded scratchpads) visualized by smFISH. This strategy is a good tool to address the lineage dynamics of a cell population. **b** In vivo lineage mapping using CRISPR/Cas9 is based on multiple integration/deletion at a target site that allows the lineage trees of the different cells to be reconstructed taking into account their genomic tags (yellow cassettes). **c** Going further, the inducible Cas9 activity (blue circle) and the incorporation of transcriptomic analysis improves these mouse models, enabling cell lineages and fate mapping to be analyzed at the single-cell level

Several other strategies have been introduced in recent years, including the implementation of CRISPR technology to drive genome editing and DNA targeting (Fig. 3). The CRISPR/Cas9 was used to perform cell lineage tracing, in theory enabling the entire organism to be reconstructed at the single-cell level using RNA or DNA sequencing. MEMOIR (mutagenesis with optical in situ readout) was the first method based on CRISPR technology that produced dynamic cell records and lineage reconstruction, combining barcoded recording elements (scratchpad) altered by CRISPR–Cas9 genome editing and in situ readouts by seq-FISH RNA imaging [85] (Fig. 3a). The limitation of this system is the lower capacity to detect edited mutations relative to those that used scRNA-seq. One of the first studies to implement CRISPR for phylogenetic analyses in mice was MARC1 (mouse for actively recording cells). These mice can accumulate genomic mutations that can be used to reconstruct the lineage tree of cells based on the homing guide RNA (hgRNA) and CRISPR/Cas9 nuclease [86]. In addition, zebrafish has been the animal model used to develop LINNAEUS (Lineage tracing by Nuclease-Activated Editing of Ubiquitous Sequences) [87], in which lineage trees of zebrafish larvae and adult cells were reconstructed by combining multiple genomic mutations and transcriptome profiling (Fig. 3b). However, the first study to infer a large-scale lineage potential was called GESTALT (genome editing of synthetic target array for lineage tracing), revealing lineage relationships during germ layer patterning in zebrafish. GESTALT employed embryos carrying an array of ten different targets that created an inheritable barcode permitting cell lineage trees to be reconstructed [88]. Nevertheless, this system was not able to distinguish the spatial location of different cell types and it was restricted to early developmental studies. Subsequently, scGESTALT was developed to improve this system, combining CRISPR/Cas9 barcode editing for large-scale lineage tracing with cell characterization using transcriptomic analyses [89]. This new approach enabled cell editing to be performed at multiple points and at later developmental stages, and accordingly it has been established as a powerful tool that enables simultaneous lineage tracing and cell sequencing in vivo [90]*.* These approaches permit the single-cell clonal dynamics and the transcriptomic profile of cells to be studied (Fig. 3c). Importantly, the clonally related cells located in the same area presented similarities in their transcriptomic profile. More recently, a new mouse line has been obtained using CRISPR/Cas9 genome editing based on lineage tracing, called CARLIN (CRISPR array repair lineage tracing: [91]). This revolutionary tool can be used to modify and detect DNA sequences in single cells line, and it could even record the history of specific stimuli, including the lineage related effects of exposure to external stimuli, stress or pathogens. One of the advantages of CARLIN is the inducible activity of Cas9 to generate barcodes at embryonic and adult stages. In conclusion, several models of CRISPR/Cas9 barcoding have shown this is a powerful tool to study fate mapping. However, cell loss or weak RNA expression are among the technical problems encountered when using these methods [92]. Nevertheless, the evolution of CRISPR-Cas9 technology along the new sequencing approaches offers very useful approaches to define fate maps in complex organisms.

### Insights into the lineage tracing of neural cells from clonal methodology

**Fig. 4:**
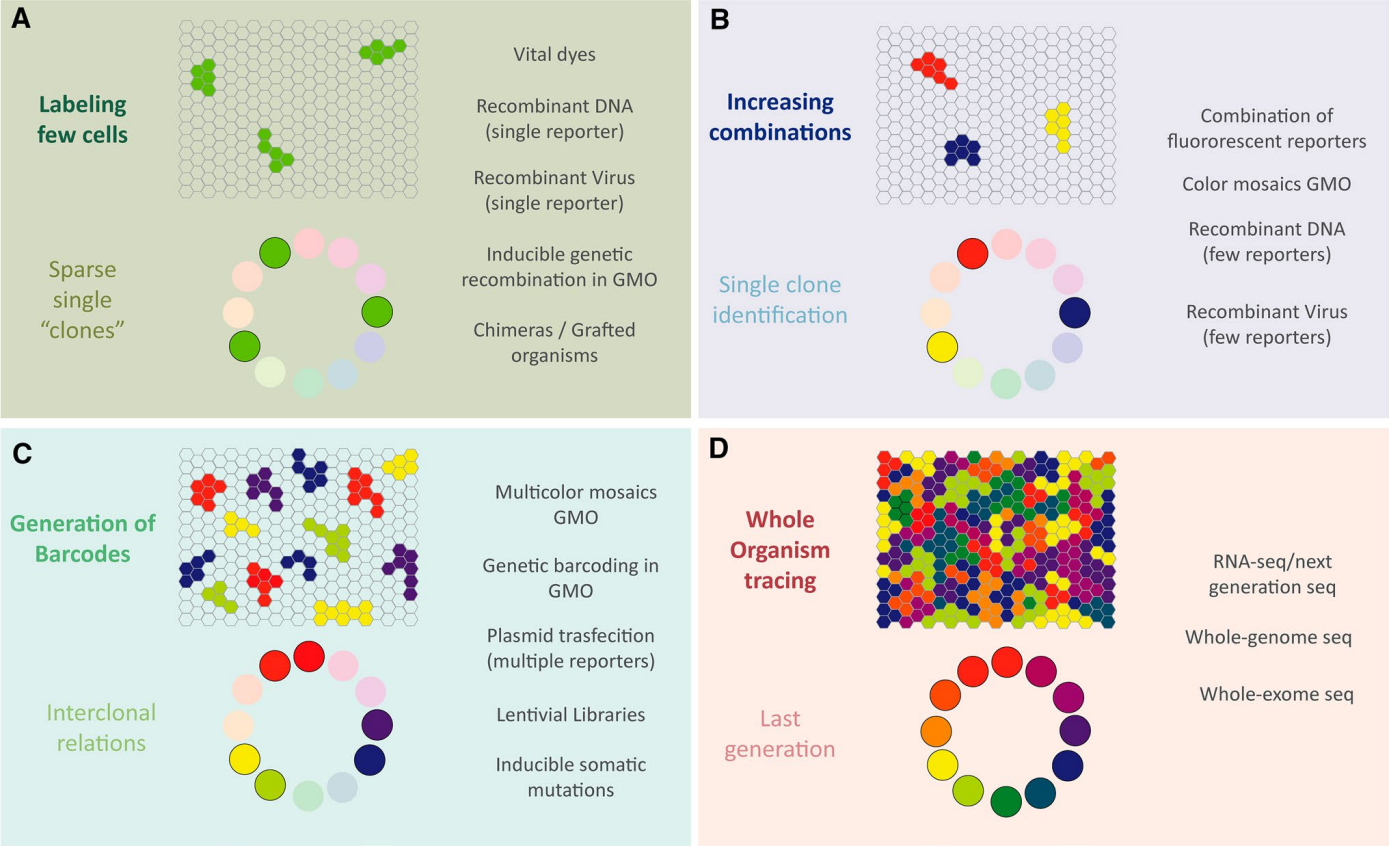
Diagram of the different techniques and their improvements based on advances in lineage tracing. **a** The first lineage analyses were done by random targeting of progenitors in a manner that attempted to ensure the distant labeling of clones. **b** The incorporation of more than one reporter increased the number of combinations and improved the single-cell clone identity. **c** The assignment of unique fingerprints to targeted progenitors enabled sibling cells to be identified and their relationships due to the generation of a specific and stable barcode in the cells. **d** However, the new tools generated to decipher genomic information in an entire organism enable a wealth of important information to be drawn that helps to reconstruct the lineage trees of cells and their sibling connections

Several methods have been proposed to examine lineage progression in different biological models in vivo or in vitro. However, their use to determine the relationships of lineages usually requires an arduous analysis. Some approaches for lineage tracing in the brain focus on the sparse labeling of cell clones to ensure the lineage connection among cells that are situated intimately in the brain (Fig. 4a). The injection of diluted viral particles to trace a minimal number of progenitors facilitates the study of the integration of neural clones into different cortical systems [93–95]. Moreover, administering low doses of tamoxifen to inducible Cre-LoxP GMOs favors the characterization of NSC potential [96]. Random Cre-LoxP recombination in sparse progenitor cells during development reveals a common origin of pyramidal neurons and astrocytes [97]. However, these methods have some drawbacks, such as the ambiguous labeling of single NPCs, the impossibility of tracing lineages that undergo diverse migratory pathways and the difficulty for inter-clonal studies. Moreover, the independent basal recombinase activity of inducible Cre lines may interfere with such tamoxifen-based lineage tracing methods [98].

Other clonal methods rely on the recombination of few fluorophores for lineage tracing (Fig. 4b), such as mosaic analysis with dual markers that have been used to determine important features of NPC potential and lineage progression in vivo [27, 99, 100]. These approaches help characterize sibling cells at a single-cell level, although only a few codes are generated for unequivocal cell identification and sparse labeling is still required.

More recently, the use of fingerprints to accurately target a single NPC and that can be inherited by their entire progeny has revolutionized the field (Fig. 4c). With these methods a larger number of clones can be assessed, allowing inter- and intra-clonal relationships to be studied, as well as identifying clonal relationships within long-distance migratory cell populations, such as adult generated interneurons [11]. Progenitor barcoding can be achieved by expressing multiple FRs, retroviral libraries or with genetic tags, such as those assembled by the CRISPR-Cas9 or Polilox system [101]. The huge improvement in sequencing technologies and genome editing tools over recent years has enriched the lineage tracing field. These techniques enable phylogenetic lineage trees to be created and linked to transcriptomic information at a single-cell level. The developing central nervous system (CNS) contains many spatially segregated germinal zones, with progenitors that generate distinct cell types. The fate of embryonic progenitors lining either the ventricular surface or the ependymal layer of the developing brain can be traced, and post-natal or adult NPCs in the brain can be also included in the lineage tracing analysis. Clonal methods have demonstrated that these progenitors may be committed to a specific cell lineage or that they may give rise to more lineages. RGCs are considered the major progenitor cell type throughout the CNS. However, new sub-types of NPCs occupy the SVZ, such as apical or basal RGCs, or apical and subapical IPCs [102]. Lineage tracing has shown that RGCs produce glial cells through IPCs or astrocytes by direct transformation [103, 104]. In addition, embryonic RGCs give rise to sibling ependymal cells and adult NSCs that remain in the lateral ventricles [105]. Postnatal NPCs have varied multipotentiality, which differs from that of restricted progenitors specified to only generate a determined lineage, or of bi-potent NPCs that generate sibling cells of two different lineages [11].

Single-cell analyses has provided huge advantages when studying cell heterogeneity and cell dynamics in NSC populations. Transcriptomic approaches have recently shed new light on lineage progression and progenitor potential, and single-cell transcriptomic analysis has revealed important information about cell identity and the distinct expression patterns under different conditions that could be crucial to fine-tune lineage tracing approaches. Transcriptomic analyses have helped classify cells in terms of their specific transcriptomic expression. Several studies have shown the diversification of transcriptional profiles within neuronal [106, 107], astroglial [108, 109] and oligodendroglial populations [110, 111]. Recent approaches using in situ transcriptomic analyses reflect the strong specification and regionalized distribution of astrocytes within the pallial cortex, diverging from the classic six neuronal layered patterns [112]. In addition, bulk RNA-seq has provided important information about gene expression and the molecular differences between cells populating neocortical layers [113]. Furthermore, pseudotemporal alignment of the transcriptomic profiles of developing cerebral organoids gave insight into the maturation and differentiation stages associated with cell-type specification [114]. The regional identity of specific neuronal progenitors has also been described for glial lineages, where progenitors in different domains produce glial cells restricted to a specific region [13, 115], with broad variability in terms of their spatial and clonal organization [116]. Thus, it becomes crucial to determine how embryonic development influences cell fate heterogeneity.

In addition, deep genome sequencing has made it possible to trace the lineage of every single cell in a given organism (Fig. 4d). DNA replication that occurs before cell division produces somatic mutations that do not have phenotypic effects. However, these somatic DNA mutations accumulate in daughter cells and they provide important information that could be useful to reconstruct lineage trees [117, 118].

Lineage tracing techniques can also be applied in pathological situations to address the heterogeneity in terms of therapeutic responses or the different implications according to ontogeny. In this regard, clonal responses have been described in the progression of squamous carcinomas [119], sarcomas [120] and breast cancers [121]. In the CNS, glioblastoma cells engage in clonal communication based on cell–cell contact [122]. Alternatively, clonal expansion of the astrocyte lineage was evident following brain disease or insult, as seen for Huntington’s disease [123], brain injury [124] or multiple sclerosis [125]. This clonal response could be explained by preferential connectivity of sibling astrocytes relative to their neighboring cells [126]. Therefore, fate mapping and cell fate potential mapping could provide important information about lineage trees and evolution, yet it could also shed light on important therapeutic issues or even, on the possible reprogramming of cells. One challenging issue will be to compile and compare all the lineage tracing data obtained through different clonal methodologies to obtain an overview of how lineages evolve [127]. All the emerging data, along with the improved molecular techniques and the developments in big data analysis, can overcome the current limitations to understand the tremendous heterogeneity among cell lineages.

In conclusion, DNA barcoding strategies and sequencing resources could help to consolidate cell lineage reconstructions and contribute to our understanding of cell dynamics, clonal expansion, and the behavior of specific cell lineages in normal conditions and disease. Moreover, this type of analysis may produce important advances in understanding the molecular events underlying lineage specification and the sculpting of progenitor potential.
